# Absorbing and transferring risk: assessing the impact of a statewide high-risk-pregnancy telemedical program on VLBW maternal transports

**DOI:** 10.1186/1471-2393-6-11

**Published:** 2006-04-04

**Authors:** David W Britt, Janet Bronstein, Jonathan D Norton

**Affiliations:** 1 Fetal Medicine Foundation of America, 131 East 65th Street, New York, USA; 2 School of Public Health, The University of Alabama at Birmingham, Birmingham, USA; 3 Department of Obstetrics and Gynecology, University of Arkansas for Medical Sciences, Little Rock, USA; 4 Department of Statistics, Florida State University, Tallahassee, USA

## Abstract

**Background:**

Prior research has shown that resources have an impact on birth outcomes. In this paper we ask how combinations of telemedical and hospital-level resources impact transports of mothers expecting very low birth weight (VLBW) babies in Arkansas.

**Methods:**

Using de-identified birth certificate data from the Arkansas Department of Health, data were gathered on transports of women carrying VLBW babies for two six-month periods: a period just before the start of ANGELS (12/02-05/03), a telemedical outreach program for high-risk pregnancies, and a period after the program had been running for six months (12/03-05/04). For each maternal transport, the following information was recorded: maternal race-ethnicity, maternal age, and the birth weight of the infant. Logistic regression was used to assess the relationship between the predictors (telemedicine, hospital level, maternal characteristics) and the probability of a transport.

**Results:**

Having a telemedical site available increases the probability of a mother carrying a VLBW baby being transported to a level III facility either before or during birth. Having at least a level II nursery also increases the chance of a maternal transport. Where *both *level II nurseries *and *telemedical access are available, the odds of VLBW maternal transports are only modestly increased in comparison to the case where neither is present. At the individual level, Hispanic mothers were less likely to be transported than other mothers, and teenaged mothers were more likely to be transported than those 18 and over. A mother's being Black or being over 35 did not have an impact on the odds of being transported to a level III facility.

**Conclusion:**

Combinations of resources have an impact on physician decisions regarding VLBW transports and are interpretable in terms of the capacity to diagnose and absorb risk. We suggest a collegial review of transport patterns and birth outcomes from areas with different levels of resources as a vehicle for moving the entire system of care forward over time. With such an evidence-based review in place, the collegial relations among level III specialists and obstetricians from around the state can, over time, develop workable protocols for when and how level III facilities should be involved.

## Background

There have been numerous studies relating the level of hospital resources to birth outcomes. Analyses of California hospital discharge data [1,2], for example, have shown that neonatal intensive care unit (NICU) level and the volume of patients in the NICU are inversely related to risk-adjusted mortality for very low birth weight (VLBW) infants, i.e., those who were born weighing less than 1500 grams. Samuelson et al reported similar findings for a very low birth weight population in Georgia, with the highest death rate reported at hospitals with the lowest level of care [3]. The same patterns have been reported in the Vermont Oxford Network [4] and Dooley et al have shown for a sample of 97 non-tertiary hospitals in Illinois that when controlling for maternal social-behavioral risk, the rate of VLBW births in such facilities is strongly associated with the hospital's fetal death rate and neonatal mortality rate [5].

From such studies it is clear that hospital resources matter when high-risk deliveries are being considered. The mechanism by which this works seems plausible and straight forward: resources concentrate diagnostic and treatment capability (both before and after birth) where they can have the greatest impact. This parallels work on co-management that suggests that high-risk-pregnancy specialists have the greatest impact on birth outcomes when they are called in before 20 weeks GA on high-risk cases [6]. The central issue is how physicians with access to different resources become sensitized to and deal with higher levels of risk. A related issue is the role that maternal characteristics have in transports [7]. In this paper we explore the impact of telemedical and hospital-level resources on the sensitization, absorption and transfer of risk with respect to the transport of mothers expecting VLBW babies in Arkansas, and we control for a variety of maternal characteristics.

Arkansas is a state in which virtually all of the high-risk-pregnancy resources are concentrated in a single place, Little Rock, the site of the University of Arkansas for Medical Sciences (UAMS) and Baptist Hospital. Located in the center of the state, it is the only city in which Maternal Fetal Medicine specialists work and the only city (with one exception, an ANGELS-supported genetic counselor in Washington County) in which prenatal-care-focused genetic counselors are present. ANGELS (Antenatal and Neonatal Guidelines, Education and Learning System) is a multifaceted educational and consultation effort designed to improve care for women with high-risk pregnancies.

Arkansas is also located in a region of the country that has had relatively high VLBW rates [8], and in a state in which VLBW rates are both relatively high and discrepant, with the rate for black mothers (3.3%) being almost 3 times the rate for white mothers(1.2%) [9]. In a proactive attempt to reduce low birth weight (LBW) rates in Arkansas, Arkansas Medicaid has funded the ANGELS-program roll out, and also provided for physician payments via telemedicine (TM) and increased the range of Medicaid coverage to 180% of the poverty line [10].

Before ANGELS existed, there was already a statewide system for developing collaborative relationships among specialists in Little Rock and physicians delivering babies around the state. These collaborative relationships go back at least as far as 1983 when an 800 number to a high-risk clinic at UAMS was started and 14 fax machines were distributed to hospitals around the state [11]. High-risk patients, then, could be transported for observation, for delivery, for post-partum surgery, for consultation regarding genetic anomalies, and on top of those possibilities, physicians could call and talk to a specialist. Further, there were limited telemedical facilities available through the Rural Health Program for a few years before ANGELS started [12,13]. If it is the case that resources shape levels of comfort that physicians feel and the decisions they make, then different combinations of resources should lead to different patterns of referral for the kinds of risk represented by mothers carrying VLBW babies.

Local resources are of two types. The first type is the level of the hospital that is available in the local community, where hospital level serves as a proxy for technological, physical and personnel resources associated typically with hospital level [14]. With the exception of Washington County in the Northwest of the state, Pulaski County (where Little Rock is located) is the only county with level III hospitals available (at UAMS and at Baptist Hospital), but there are several level II hospitals scattered around the state, and several more communities in which only a level I hospital is available. Level II hospitals can diagnose and handle more complex problems – i.e., absorb more risk – than level I hospitals, and level II nurseries can typically care for neonates born as early as 32 weeks [15]. Further, the staff members at such facilities see more high-risk cases and have a better sense of what they can safely handle and what should be transported.

Telemedical facilities linking level I and level II facilities to UAMS's tertiary care center are another type of local resource. They enable sharing of information regarding patients and treatment options in real time between a local physician and a specialist at UAMS. Hence, where such facilities are available, there should be a greater capacity to understand locally risks associated with different pregnancy complications. In contrast to hospital resources, however, telemedical resources are both local and collective in that they *link *the higher-level resources of a level III facility with the lower-level resources available at level I and level II facilities.

Our expectations for the implications of different combinations of telemedical and hospital-level resources on the chances of transport of mothers carrying VLBW babies are based on the assumption that the meaning of these resources is dependent on the value of the other (Table 1). Doctors in areas with access to neither telemedical nor level II hospital resources (denoted **la **in Table 1) may not see as many high-risk pregnancies and may be poorly equipped to assess the level of risk and treatment options. Physicians in such under-resourced areas may also be relatively unaware of the nature of specialist resources and/or reluctant to contact specialists whom they barely know. Hence, mothers in such areas carrying VLBW babies may not have a very good chance of being transported to a level III facility in Pulaski or Washington County. For this study, these low-resource areas are the baseline condition against with other combinations are assessed.

**Table 1:** Defining resource contexts

	Level I Hospital/Nursery (**l**)	Level II Hospital/Nursery (**L**)
Low Telemedical Access (**a**)	**la**	**La**
High Telemedical Access (**A**)	**lA**	**LA**

Both telemedical and level II resources increase access of local physicians to expertise regarding high-risk pregnancies and experience in dealing with them. Two immediate consequences flow from this. Sensitive to variation in these resources is the development of a collegial dialog regarding the nature of risk, treatment options and the limits of local care. Where telemedical resources are present, but not a level II nursery (**lA**), the collegial dialog involves local obstetricians and other physicians with one another (should others be present) and dialog with specialists at UAMS either by phone or telemedically (with and without the patient present). On the basis of an earlier study of the preliminary organizational impact of ANGELS, we know that levels of telemedical and phone communication between obstetricians around the state and specialists at UAMS significantly increase [16]. Where there is no telemedical access, but there is a level II facility (**La**), the chances are increased that this dialog or communication routine will involve local pediatricians and other medical personnel who have experience in high-risk care. UAMS specialists are available by phone, if need be. The capacity to effectively treat locally high-risk pregnancies increases. For VLBW situations, it is the latter aspect that comes in to play more quickly. Hence, we expect that with exposure to *either *of these resources that the chances of transport for a woman carrying a VLBW baby increase.

On the other hand, with access to *both *the resources of a level II facility *and *the expertise of UAMS specialists via telemedicine (**LA**), the sense of being able to handle the contingencies associated with VLBW increase. Hence, we expect the chances of a transport to decrease from what would be expected if the resources were operating separately.

## Methods

This Institutional Review Board-approved study took place at the University of Arkansas for Medical Sciences' Department of Obstetrics and Gynecology. The specific program examined was the ANGELS telemedical system. The associated high-risk pregnancy clinic has a 24-hour 800 number and is staffed by 4 Maternal-Fetal Medicine specialists and, in addition, associated residents. The nurseries at UAMS consist of a 32 bed NICU and a 20 bed Intermediate Care nursery. Each year the facility hosts approximately 1800–2000 deliveries and serves as a high-risk tertiary-care facility that receives referrals from level I and level II facilities in almost all of Arkansas' 75 counties (71 in fiscal year 2003–2004), as well as bordering counties in neighboring states. During the 6-month period before ANGELS started, there were 5 operational telemedical sites (a legacy of the Rural Health Program), and during the 6-month period after ANGELS started, there were 9 active telemedical sites. (A total of 13 sites were in place during the post-ANGELS period, of which 9 were in active use. The analysis below is based on the active sites.) The sites were equipped with two-way compressed video and connected via T1 lines. A typical site included a TANDBERG Intern II tele-healthcare unit (TANDBERG, New York, USA), an ALI store-and-forward system (McKesson Information Solutions, Richmond, Canada), and a portable ultrasound machine.

Data were gathered from the birth certificate forms for the years 2002–2004. The data were sent to the investigators in de-identified form, i.e., they did not include items such as name, exact birth date, or Social Security number that would allow the investigators to uniquely identify an individual. For each transport of a VLBW-baby-carrying maternal transport, the following information was provided: birth weight, mother's race/ethnicity, and maternal age. From ANGELS' administrative records, the degree of access to a telemedical site was operationalized as being at least adjacent to a county with a telemedical facility. From Arkansas Department of Health data, the highest level hospital in the mother's county of residence, where transfer is presumed to have originated, was recorded.

We consider transports of mothers who give birth to VLBW babies when they are transported *before *they give birth and when both they and their babies are transported. Excluded from analysis are baby transports after they are born. We examine only those Arkansas counties that are not touching Pulaski County, where both UAMS and Baptist Hospital are located. These counties that form a belt around Pulaski County have a special relationship with UAMS with an agreement that UAMS will do all the deliveries if the county medical units do the prenatal care. For the counties outside of this belt, there were 21 maternal-only transports and 70 maternal-and-child transports out of a total of 415 VLBW babies. Our sample, then, consists of 415 VLBW-baby-carrying mothers, 91 (21.92%) of whom were transported to a level III facility.

In order to test the effect of the various predictors on the odds of transport, we performed logistic regression using SAS 9.1 software (SAS Institute Inc., Cary, NC). The Wald X^2 ^test was used to assess the significance of individual predictors. Odds ratios for transport were estimated for predictors in the final model, and confidence intervals were computed using a normal approximation. Table 2 presents the marginal percentages for the variables included in the logistic regression analysis.

**Table 2:** Marginal percentages for variables used in the regression analysis

	N	Marginal Percentage
Maternal transport		
0	324	78.1%
1	91	21.9%
Telemedical site availability		
0	188	45.3%
1	227	54.7%
Teen mother		
0	381	91.8%
1	34	8.2%
Hispanic mother		
0	387	93.3%
1	28	6.7%
Level II Nursery		
0	200	48.2%
1	215	51.8%
Telemedical site and Level II Nursery		
0	257	61.9%
1	158	38.1%

Total	415	

## Results

We initially tested models that had only additive effects for telemedical access and hospital level and a variety of individual-level controls, including advanced maternal age (> 35), teen mother (< 20), Hispanic mother and Black mother. In none of these analyses were Black mother and advanced maternal age close to being significant. They were dropped from the final model. Table 3 presents the results of our final logistic regression analysis, which includes a pre-specified interaction between the effects of hospital level and telemedicine access.

**Table 3:** Logistic regression results for VLBW maternal transports*

Variable	B	Standard error	Significance	Odds Ratio	Lower bound of 95% CI	Upper bound of 95% CI
Intercept	-1.68	.24	< .001			
TM (telemedical site availability)	.98	.35	.005	[given low hospital resources] 2.66 [given high hospital resources] (.55)	1.34 (.39)	5.30 (.79)
Ob2 (level II hospital)	.81	.38	.032	[given low telemedical access] 2.25 [given high telemedical access] (.47)	1.07 (.24)	4.71 (.89)
TM*Ob2	-1.57	.50	.002			
Teen mother	.84	.39	.032	2.31	1.07	4.98
Hispanic mother	-1.44	.76	.057	.24	.05	1.05

* Because of the interaction, the OR's for TM and Ob2 depend upon the value of the other variable. The estimates not in parentheses represent the estimate of the OR for the given predictor with the value of the other variable at "0." The OR estimates in parentheses represent the estimate with the other variable at "1."

Let us present the control variable results first. Teenaged mothers are more than twice as likely to be transported as non-teenaged mothers (OR = 2.31, p < .05). Conversely, Hispanic mothers are apparently less likely to be transported than non-Hispanic mothers (OR = .24, p = .06).

The main thrust of our analysis focuses on the implications of the access of mothers and their physicians to different combinations of telemedical and hospital-level resources. Because of the interaction effects involved, these results must be presented conditionally, with the results for each resource type being presented within levels of the other resource type. Each resource type, in effect, provides context for the transport-related impact of the other. With respect to telemedical access, when such access occurs in the context of level I hospital resources or less, such access more than doubles the odds of mothers carrying VLBW babies being transported (OR = 2.66). When telemedical access occurs in the context of at least level II hospital resources, on the other hand, the odds of mothers being transported are reduced relative to what would be expected if the resources were making independent contributions. This interaction between the effects of telemedicine and local hospital level can be seen in two ways. One way is to look at the effect of telemedicine, relative to no telemedicine, in the presence of level II or higher resources. This gives a conditional odds ratio of .55, as shown in Table 3. Alternatively, one can assess the joint effect of a facility having telemedicine access and level II or higher resources, compared to having neither. The odds ratio for transport in the presence telemedical access *and *level II hospital resources, *relative to the case where both of these elements are absent*, is 1.25.

The results are essentially the same for hospital level. When increased hospital-level resources are available in the context of no telemedical access, the odds of mothers carrying VLBW babies being transported are considerably increased (OR = 2.25). In the context of telemedical access, however, the conditional odds ratio associated with increased hospital level is 0.47. Again, this interaction may also be understood by looking the odds ratio with both level II obstetrics and telemedicine being present compared to both being absent (OR = 1.25).

## Discussion

The main focus of this paper is the impact of different resource combinations. The individual-level controls deserve some discussion beforehand. Relative to white (and other) women between the ages of 18 and 34, teenaged women were more likely to be transported and Hispanic women were less likely to be transported, while black women were no different in their chances of being transported. Teenaged mothers with no prior pregnancies may be adjudged to be at greater risk [17]. Hispanic women, on the other hand, may be adjudged to be at less risk. In Arkansas and in the rest of the country, Hispanic women have been shown to be at less risk of having a LBW baby [18]. These individual-level results need to be studied further with data that permit identification of mothers in order to rule out the possibility that Hispanic mothers just do not have the same access to care that others do, or that teenagers are not referred for other reasons. It should be noted, however, that only 6.7% of the VLBW-carrying mothers in our sample (Table 2) were Hispanic. Hence, even with significant improvement in the odds of transport for Hispanic mothers, there would only marginally be changes in the odds of VLBW-carrying mothers being transported to a level III facility.

The resource-combination results lend support to the assumption that resources are critical to the decisions physicians and local clinics make about pregnancy risk, and to the companion assumption that *combinations *of resources form substantively different contexts within which physicians operate. These results also lend support to the more general assumption that contexts are important [19], and they show the efficacy of looking at more complex systems rather than individual treatment – a key challenge for health protection research [20].

Before discussing these results in greater detail, it is necessary to speak to the possibility of alternative explanations for our results. Were it the case that existing ANGELS sites have been placed in wealthier areas where there are better staffed, larger hospitals – areas that have better resources and serve patients on average with better resources – then this might constitute an alternative explanation for our results. Such is not the case. In a previous paper we examined the impact of several county-level factors on the odds of ANGELS site placement [10]. Neither county poverty levels nor the county ratio of black to white births had a statistically-significant impact on the odds of site placement. With two county-level characteristics – the log of births and a history of above-median LBW births – a model was developed that satisfactorily discriminated between those counties with and without sites. The same results were found for a model that also included the ratio of black to white births. The area under the ROC curve was .84 for both models. These results are pertinent because they signify that site placement has followed need and risk rather than convenience and resources. Consequently, an alternative explanation based on bias having been introduced into sites or hospital selection is less tenable.

It might also be argued that it is not possible to separate out the effects of the ANGELS program as a whole from the effect of sites. Specifically, there have been improvements in ANGELS' back-office procedures as well as the development of specific telemedical sites. We have had to make the simplifying assumption that such improved back-office procedures affect all areas equally, so that differences between resource combinations (telemedicine, hospital level) and the baseline condition are attributable to the resources themselves. Future research should be conducted with multi-method designs that permit measuring and separating out these kinds of differential effects on quality of care indices.

## Conclusion

The practical importance of the patterns we have observed in these different contexts is potentially great owing to the early stages of development of the ANGELS telemedical system. Of the VLBW-carrying mothers in our sample, almost a third (31.56%) had access to neither a telemedical site nor a level II nursery facility (our baseline comparison group). Our coding scheme for telemedical site availability gave a high score to being in a county that touched a county that had telemedical access, so even though 54.7% of the mothers in our sample had such access, in many cases the distances needed to travel might have been up to 50 miles.

We believe that both telemedical and local hospital resources *separately *engage physicians in a more collegial communication routine regarding risk and treatment options, with consequent increases in high-risk transports. When both of these types of resources are *jointly present*, however, they appear to lead to a greater absorption of the risks associated with VLBW delivery. Since the quality of care available at level III facilities is higher than that at other facilities, what this means in practical terms is that areas with no resources of either kind and areas in which both telemedical and level II hospital resources are present are in need of dialog and training regarding what it is appropriate for them to handle and how early specialists at level III facilities should be involved.

There is not a definitive policy recommendation regarding how local and remote care providers should coordinate their behavior, however. Such knowledge can only come from examining fetal, neonatal and perinatal mortality for these areas over time and how these outcomes are linked to decisions regarding transports. What we recommend is an *evidence-based yearly review *of birth outcomes associated with transfer patterns from areas with different combinations of resources. Such a policy feedback process would be a system-level analog to the collaborative information sharing mechanism that we believe is important in understanding VLBW transport patterns by context. It is also compatible with the "obstetric consultation and privileges guidelines" described by Beerman et al for comanagement within a single, research-intensive academic medical center. In such a system, who should be doing what with respect to diagnosis, treatment and delivery is formally defined [21].

Moving beyond the geographically narrow setting in which all participants are either residents or faculty in the same medical school as specified by Beerman et al to a loose, mostly informal set of relationships among physicians around an entire state requires the development of a less formalized, more negotiated solution – one based on evidence regarding what outcome/transfer-decision pairings from areas with different resource characteristics. An evidence-based, collegial examination of the birth-outcome implications of different transfer patterns from areas with different resource combinations is the sort of "system" that Stange has suggested for developing a shared-care mix that optimizes patient outcomes [22].

As in the system that Stange envisions, such evidence-based negotiations regarding appropriate transfers should lead over time (a proxy for review cycles) to a mutually satisfying definition of roles and responsibilities and an explicit understanding of each other's roles [23]. Where a statewide system is being discussed, however, there would be a critical change in the analysis and negotiations. For smaller, less heterogeneous systems, the following observation by Stange seems reasonable:

"... [T]he best mix of specialist and generalist care is still not clear, but it is likely to partially depend on the skill of the primary care physician in offering optimal first-line diagnosis and treatment."[23]

A larger, more diverse system, both in terms of geography and resources, must shift from a discussion in terms of "the skill level of the primary care physician" to a consideration of the practical implications and typical birth outcomes associated with areas with different levels of resources.

Although this is a broader review than Strange and others have suggested, it is not an abstract and academic recommendation. Indeed, it is oriented toward a periodic review of the mechanics of what works and does not work in areas with different kinds of resources. All systems break down on occasion, and some way of reviewing these breakdowns is essential. For obstetricians working around the state, breakdowns might take the following form, as described by one physician with whom we talked:

(paraphrased) What happens to us *a lot *(physician emphasis) is that even if we refer a high-risk patient down to UAMS, and even if UAMS's intention is to follow up with that person and eventually deliver her, it is *very common *for that woman to walk into our back door when she is in the act of labor, and there is no time to transfer and you have to deliver the patient, afterwards getting ANGELS involved to transport the baby to ACH [Arkansas Children's Hospital].

Such "backdoor" breakdowns may be preventable through an anticipated internet-based monitoring system for all pregnant women after their first contact with a physician, but there are other kinds of breakdowns that are resolvable only through continuing dialog, education, protocol development and coordination.

Only about 22% of VLBW babies in our sample involved maternal transports, and a sizeable percentage (32%) of VLBW-carrying mothers (and presumably their doctors) were located in areas with access to neither telemedical nor level II nursery resources. Physicians in such areas should be prime targets for follow-up dialog and attempts to involve them in the network, since from a best-practice point of view they should be the most likely to transport VLBW-carrying mothers rather than the least likely. Such an approach is system-oriented rather than individual-risk-oriented.

## Competing interests

The author(s) declare that they have no competing interests.

## Authors' contributions

All three authors collaborated on the design of the study. JN proposed and carried out the statistical analysis. DB conducted the physician interviews which motivated the study, including the interview cited in the text. JB collaborated in the statistical design and the interpretation of the transport and interview data. DB was the primary writer of the manuscript, but the other two authors were actively involved in its production and approved the final version.

## Pre-publication history

The pre-publication history for this paper can be accessed here:


